# Injection of cocaine is associated with a recent HIV outbreak in people who inject drugs in Luxembourg

**DOI:** 10.1371/journal.pone.0215570

**Published:** 2019-05-16

**Authors:** Vic Arendt, Laurence Guillorit, Alain Origer, Nicolas Sauvageot, Michel Vaillant, Aurélie Fischer, Henri Goedertz, Jean-Hugues François, Ivailo Alexiev, Thérèse Staub, Carole Seguin-Devaux

**Affiliations:** 1 Service National des Maladies Infectieuses, Centre Hospitalier de Luxembourg, Luxembourg, Luxembourg; 2 Department of Infection and Immunity, Luxembourg Institute of Health, Esch sur Alzette, Luxembourg; 3 National Drug Coordinator, Ministry of Health, Luxembourg, Luxembourg; 4 Competence Center for Methodology and Statistics, Luxembourg Institute of Health, Strassen, Luxembourg; 5 Clinical and Epidemiological Investigation Center, Luxembourg Institute of Health, Strassen, Luxembourg; 6 Stop AIDS Now Access, Luxembourg, Luxembourg; 7 Molecular Biology Laboratory, Centre Hospitalier de Luxembourg, Luxembourg, Luxembourg; 8 National Reference Laboratory of HIV, National Center of Infectious and Parasitic Diseases, Sofia, Bulgaria; University of Cyprus, CYPRUS

## Abstract

**Background:**

An outbreak of HIV infections among people who inject drugs (PWID) started in 2014 in Luxembourg.

**Objectives:**

We conducted phylogenetic and epidemiological analyses among the PWID infected with HIV in Luxembourg or attending the supervised drug consumption facility (SDCF) to understand the main causes of the outbreak.

**Methods:**

Between January 2013 and December 2017, analysis of medical files were performed from all PWID infected with HIV at the National Service of Infectious Diseases (NSID) providing clinical care nationwide. PWID were interviewed at NSID and SDCF using a standardized questionnaire focused on drug consumption and risk behaviours. The national drug monitoring system RELIS was consulted to determine the frequency of cocaine/heroin use. Transmission clusters were analysed by phylogenetic analyses using approximate maximum-likelihood. Univariate and multivariate logistic regression analyses were performed on epidemiological data collected at NSID and SDCF to determine risk factors associated with cocaine use.

**Results:**

From January 2013 to December 2017, 68 new diagnosis of HIV infection reported injecting drug use as the main risk of transmission at NSID. The proportion of female cases enrolled between 2013–2017 was higher than the proportion among cases enrolled prior to 2013. (33% vs 21%, p < 0.05). Fifty six viral sequences were obtained from the 68 PWID newly diagnosed for HIV. Two main transmission clusters were revealed: one HIV-1 subtype B cluster and one CRF14_BG cluster including 37 and 9 patients diagnosed since 2013, respectively. Interviews from 32/68 (47%) newly diagnosed PWID revealed that 12/32 (37.5%) were homeless and 27/32 (84.4%) injected cocaine. Increased cocaine injection was indeed reported by the RELIS participants from 53 to 63% in drug users with services contacts between 2012 and 2015, and from 5 to 22% in SDCF users between 2012 and 2016. Compared with PWID who injected only heroin (n = 63), PWID injecting cocaine and heroin (n = 107) were younger (mean of 38 vs 44 years, p≤0.001), reported more frequent piercing (≤0.001), shared and injected drugs more often (p≤0.01), and were more frequently HIV positive (p<0.05) at SDCF using univariate logistic regression analysis. Finally, in the multivariate analysis, use of heroin and cocaine was independently associated with younger age, piercing, sharing of drugs, and regular consumption (p<0.05).

**Conclusions:**

Injecting cocaine is a new trend of drug use in Luxembourg associated with HIV infection in this recent outbreak among PWID.

## Introduction

Latest estimates report around 15.6 million people who inject drugs (PWID) worldwide [1]. In Europe, newly reported cases of HIV among PWID remained stable in recent years[2]. Nevertheless, several large local outbreaks were described in Europe and, more recently, in the United States, which demanded effective scaled-up prevention responses [3–8]. These outbreaks appeared in specific conditions that foster rapid spread of the virus such as economic crisis, increased homelessness, or no access to needle/syringe exchange programs (NSP) and HIV testing [9].

In Europe, PWID accounted for approximately 14.0% of new HIV-diagnosed cases in 2014 [10] with various distributions ranging from 3.1 in Western Europe to 27.8% in Eastern Europe [11]. Combination of harm reduction programs with HIV testing and councelling, antiretroviral therapy and condom distribution are required for effective prevention against HIV in this target population [9]. In Luxembourg, the prevalence rate of PWID was estimated at 3.77 per 1000 persons aged 15–64 years providing an estimated number of 1,550 PWID in 2017 [12]. Access to opioid substitution treatment (OST) is fairly high with around 1,200 registered OST patients nationally. More than 200 users daily attend the national supervised drug consumption facility (SDCF) [13]. Both OST and national NSP are well developped, decentralised, and available in prison. Intravenous heroin and cocaine use is the predominant pattern for primary use associated to poly-drug use in combination with crack or free base cocaine, speedball, crystal meth or LSD. Injecting cocaine has becoming increasingly popular in recent years [14]. A rapid increase in cocaine supply has indeed been reported in Luxembourg in drug users from the SDCF early 2011 in parallell with seizures of cocaine, changing the drug market landscape and restricting access to heroin.

**Fig 1:**
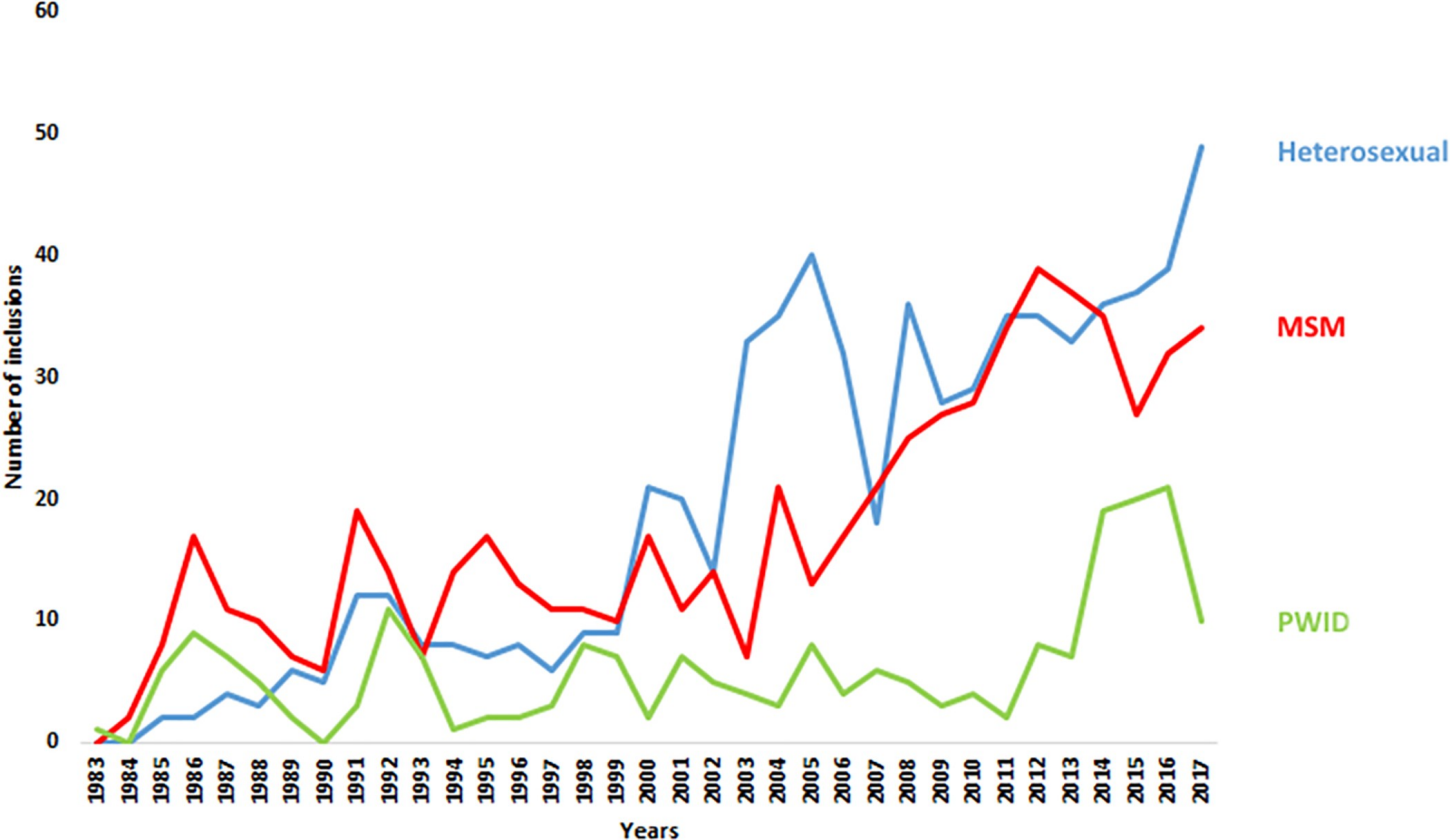
Number of HIV-infected patients newly enrolled at the “Service National des Maladies Infectieuses” by year from 1983 to 2017 according to risk group. MSM, Men who have Sex with Men. PWID, People Who Inject Drugs.

Since the beginning of the epidemic, all newly diagnosed HIV-infected patients are referred to the National Service of Infectious Diseases (NSID) at the Centre Hospitalier de Luxembourg. Up to December 2017 a total of 1,679 patients infected with HIV were enrolled at the NSID. Despite a high coverage of antiretroviral therapy (88%) among people diagnosed with HIV and high rates of virologic suppression (92%) in ART-treated patients in Luxembourg [15], the number of all HIV cases referred at NSID in 2017 has doubled since the early 2000s (Fig 1) with a marked increase from 2011 to 2017. The majority of all HIV/AIDS cases has been attributed to heterosexual and male homosexual transmissions. Between 2011 and 2014, HIV prevalence in PWID varied between 2 and 6% in the whole country [14]; HIV transmission within PWID then rapidly expanded. The HIV prevalence rate in PWID was estimated at 13% in 2016, and Luxembourg reported the highest rate of newly diagnosed HIV cases attributed to injecting drug use in Western and Eastern Europe (33 cases per million population) [14]. The main objective of the present study was to investigate the potential causes of the HIV outbreak observed among PWID between 2012 and 2017.

## Materials and methods

### Study sample

Three different methods for data collection were used in this study to investigate the HIV outbreak: (1) a review of HIV diagnoses during 2013–2017 at the NSID including HIV sequences for phylogenetic analysis, (2) an analysis of the RELIS database from 2012 to 2016, and (3) interviews and HIV, HBV, HCV testing performed at SDCF during October 2015 to December 2017 in an outreach program.

A retrospective study was first conducted on all HIV-infected patients diagnosed between January 2013 and December 2017 at the NSID of Luxembourg, located at the Centre Hospitalier de Luxembourg, in charge of the clinical follow-up of all HIV-infected patients in the country. All persons with confirmed HIV diagnoses are referred to NSID. During the investigation of the HIV outbreak, baseline data prior ART initiation were extracted from medical records of all newly diagnosed patients providing written inform consent when they were referred to NSID (study approved by the National Research Ethics Committee of Luxembourg, CNER 2013-04-22). Viral load, CD4 cell counts and sequencing of PRO-RT genes were routinely performed in the first blood sample at baseline when patients were naive to antiretroviral therapy (VL above 1 000 cp/mL) using Viroseq v2.0 (Abbott, Germany) on an ABIprism 3500 xl IVD sequencer (Applied Biosystems, Belgium).

An outreach program was initiated in October 2015 to further expand testing among PWID at SDCF. The study was approved by the National Research Ethics Committee of Luxembourg (CNER 2014-07-11). All participants provided written informed consent. Any adults having injected once any illicit drugs were recruited. HIV-seropositive individuals, whose diagnosis was confirmed, were further referred to NSID, as for other testing centers, and baseline data prior to ART initiation were extracted up to december 2017 including routine baseline virological sequences for phylogenetic analyses.

### Phylogenetic analysis

HIV-1 subtypes were determined by the automated tool COMET (https://comet.lih.lu/), by recombinant analysis using jpHMM [16] and by manual phylogenetic analysis using approximate maximum-likelihood (ML) [17]. All available global HIV-1 sequences spanning the HIV-1 protease (PR) and reverse transcriptase (RT) genes (HXB2 position 2253–3450) (n = 42,517), 170 subtype reference sequences from the Los Alamos National Laboratory database (http://www.HIV.lanl.gov) together with HIV sequences of all patients followed at NSID since 1983 (1. 045 patients) were used as a primary dataset. Sequences were aligned using mafft.v7, visualized and cleaned with SeaView.v4. Phylogenetic trees were inferred using the approximate ML analysis method under the GTR+cat model of nucleotide substitution including a gamma (G) distributed rate of heterogeneity among sites, implemented in FastTree v2.1 and edited by FigTree v1.4. Confidence values of the tree topology were assessed by the Shimodaira-Hasegawa (SH) test [17]. After the initial selection, all sequences were further subjected to quality control analysis using an in house quality control tool and subtype confirmation using COMET v1.0. and jpHMM. The automated quality check followed a number of criteria: only sequences with a minimal correct base pair names and a minimal length of 1,000 bp, no evidence of hyper-mutations, stop codons and/or frameshifts, ambiguities. Duplicate sequences were identified and only one sequence per patient was used. From these initially collected sequences globally, the phylogenetically closest sequences, reference sequences and specially selected representative sequences from different regions of the world and Europe were selected. Thus, the second formed dataset of subtype B (n = 20, 928) and CRF14_BG (n = 107) sequences, respectively, were used to analyze possible phylogenetic clusters separately. Phylogenetic transmission clusters were defined as clusters if composed of at least 3 sequences and harboring an SH value ≥0.95.

### The RELIS database

The RELIS database [1] was consulted to determine the frequency of cocaine/heroin use from 2012 onwards. RELIS is a nationwide monitoring system collecting harmonized core data on drug users in contact with national drug demand reduction and drug supply reduction services. Comparable data of 600 to 700 drug users are collected routinely and anonymously on a yearly basis to edit the national drug report as well as for strategic planning, policy and research purposes. The registry is approved by the “Comité National de la Protection des Données”. Any adults in contact with national drug demand reduction and drug supply reduction services or hospitals for addiction’s treatment were recruited. All participants provided informed consent. RELIS is a monitoring system to estimate trends in the extent (prevalence, incidence) and pattern of problem drug use. Drug users might indicate if they have been tested yet for HIV, HBV and HCV. HIV testing should be crossed with drug use behaviors (injecting, needle sharing), and the testing history should be verified as far as possible by the treatment centers and the SDCF.

### Epidemiological analysis on drug consumption

An outreach program was initiated in 2015 to diagnose and interview PWID on drug consumption and risk behaviors at SDCF. The study was approved by the National Research Ethics Committee of Luxembourg (CNER 2014-07-11). All participants provided written informed consent and were tested for HIV, HBV, HCV. Any adults having injected once any illicit drugs were recruited. Interviews were conducted at SDCF (90% of participants) but also at NSID (10% of participants) (depending on the availability of the drug user) between October 2015 and December 2017 using a standardized questionnaire including: demographic and social characteristics, drug use patterns, risk and harm reduction behaviours and infectious diseases status. The refusal rate was estimated at 20% in both sites. The national SDCF is an integrated centre for legal use of intravenous drugs providing multiple services for drug users. These include safer use and risk reduction offers, provision of sterile injection paraphernalia, a clean, quiet and secure environment for supervised licit and illicit drug consumption (both injecting and blowing). An average of 1,700 users were registered annually since 2016 to use illicit drugs in a supervised way with clean material, and to be treated in case of overdose. However, SDCF provides multiple services for drug users such as an emergency night shelter located in the same building. SDCF is also the largest NSP service in Luxembourg. Opioid substitution therapy (OST) is not dispensed from SDCF to prevent overdose but OST is available from other drug treatment services or from the black market. Collection of routine programatic data are collected for all clients of the SCDF but were not used in the epidemiological analysis. The number of users per year can not be provided as for various reasons only the cumulative number of user contracts over the years and daily, weekly, monthly and yearly consumption episodes within the SDCF are registered.

### Statistical analyses

Continuous variables of the questionnaire were described with median and interquartile intervals (IQR) whereas categorical variables were described with frequencies and percentages. Differences between the group of cocaine injectors (showing concomitant heroin injection use or not) and the group of heroin injectors (without cocaine injection use) were tested with the Chi-square test, except for age and age at first consumption which were tested with the Mann-Whitney test. Difference of characteristics across groups (heroin and cocaine versus heroin only) were tested with univariate logistic regression and described with odds-ratio and their 95% confidence intervals. To assess independent relationships, characteristics significant at the 5% level in univariate logistic regressions were all introduced in a same multivariable logistic regression model. A sensitivity analysis was carried out by using a higher selection cut-off (p = 0.20) of variables in univariate analyses for the multivariable logistic regression model. All statistical analyses were performed using the SAS software version 9.3 (SAS, Cary, NC, USA). All p-values are two-sided.

## Results

Early March 2014, a higher number of 6 new HIV infections among PWID was recorded at NSID leading to a closer investigation of the new HIV cases An outreach program was further launched at the SDCF in October 2015 by clinicians of the NSID supported by research nurses of the Luxembourg Institute of Health to expand targeted HIV screening in this key population and a total of 77 PWID were referred to NSID between 2013 and 2017 (Fig 1), of which 68 PWID were newly diagnosed for HIV and 9 were previously infected with HIV in another country but not referred to NSID. Among them 21 were newly diagnosed at SDCF from October 2015 via the outreach program. Among the 68 newly diagnosed PWID cases, the median age was 36 years old (IQR 31–38), 51/68 (75%) were male, 41/68 (60%) were born in Luxembourg, 39/68 (57%) stopped school before the age of 15 years old and 54/68 (79%) were unemployed. 22/68 (32%) were diagnosed early after infection (four patients were diagnosed during the acute phase of infection and 18 seroconverted within 6 months as shown by HIV-1 western-blotting [18]) .55/68 (80%) PWID were co-infected with HCV.

**Fig 2:**
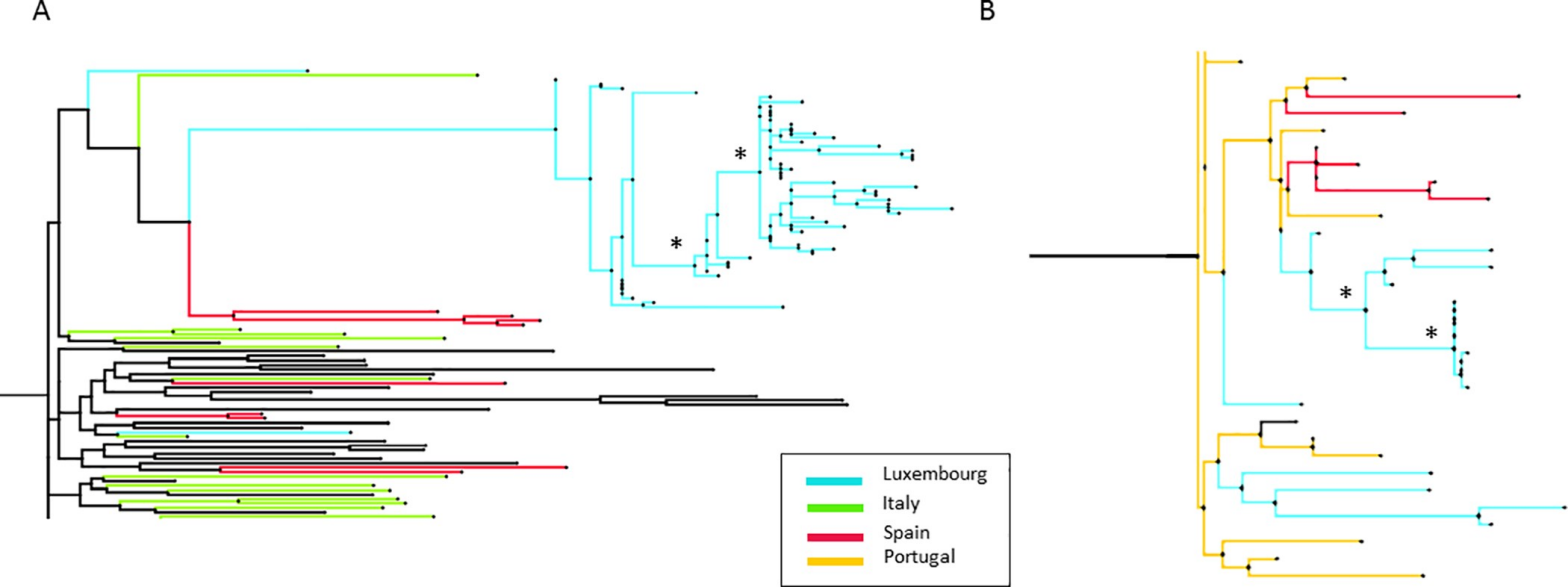
Phylogenetic trees of the two PWID HIV-1 subtype B and CRF14_BG clusters from Luxembourg related to sequences from other countries. **A. Phylogenetic tree of the PWID HIV-1 subtype B cluster. This cluster of 52 sequences from Luxembourg** are related to sequences from Luxembourg, Italy and Spain. * SH value ≥ 0.95. All other SH values in the cluster were above 0.8. Diamonds represent the node of a sequence in the tree. **B. Phylogenetic tree of the PWID HIV-1 CRF14_BG cluster of 10 sequences.** This cluster of 10 sequences from Luxembourg are closely related to sequences from Spain and Portugal. * SH value ≥ 0.95. All other SH values in the cluster were above 0.76. Diamonds represent the node of a sequence in the tree.

Fifty six baseline viral sequences (one sequence per PWID) were obtained from the 68 PWID newly diagnosed for HIV: 43/56 viruses were subtype B (77%), 9/56 CRF14_BG (16%), and 4/56 cases (7%) were either C, CRF02_AG, CRF06_cpx or URF. The phylogenetic analysis revealed two main transmission clusters among PWID. One large cluster of 52 HIV-1 subtype B sequences collected at the NSID since the beginning of the epidemic included 37 PWID diagnosed since 2013 (SH value of 0.97, Fig 2A). Another cluster composed of 10 CRF14_BG sequences included 9 PWID diagnosed between 2013 and 2017 (SH value of 0.95, Fig 2B). The B and CRF14_BG clusters orginated from two most recent common ancestors identified in Luxembourg in 2007 and 2000, respectively. The HIV-1 B cluster was closely related to Spanish and Italian sequences whereas the CRF14_BG cluster was related to Portugese and Spanish sequences These results showed network transmissions from local strains circulating among PWID.

A higher proportion of females was observed among the 68 newly diagnosed PWID as compared to the proportion of PWID females followed at NSID prior 2013 (33 vs 21%, respectively, T-test p < 0.05). The female PWID diagnosed during the outbreak described trading sex for drugs or money. Among the 68 new HIV cases, 56 (82%) individuals had ongoing or past HCV co-infection. Table 1 depicted the epidemiological and clinical data of these new cases according to clusters. PWID from the B cluster were predominately born in Luxembourg (28/37 cases) whereas the majority of the PWID who did not belong to a cluster were not natives (14/22 cases) and were mainly born in Western Europe (69%). The geographical location of the B cluster was distributed in Luxembourg City (near the SDCF) whereas all PWID from the CRF14_BG cluster live in the southern district of Esch.

**Table 1:** Epidemiological and clinical data of the 68 people who inject drugs newly diagnosed with HIV since 2013 according to clusters.

Cluster	Median age (IQR^a^)	Sexe (%)	Birth Country (%)	Median viral load at enrollment (IQR^a^) cp/mL	Median CD4 at enrollment (IQR^a^) cells/μL	HCV^b^ Infection (%)	HCV^b^ genotype (%)
Cluster HIV^c^ B n = 37	34.5 (31–38)	Male: 62.1 n = 23 Female: 37.8 n = 14	Luxembourg : 75.7 n = 28 Other countries : 24.3 n = 9	13 017 (3 065 - 31 199)	638 (466–842)	Without evidence of exposure: 5.4 n = 2 Past : 21.6 n = 8 Ongoing : 3. n = 27	G1a : 50 n = 15 G2: 3.3 n = 1 G3a : 46.7 n = 14
Cluster HIV^c^ CRF14_BG n = 9	37.5 (33.5–41.5)	Male: 88.9 n = 8 Female: 11.1 n = 1	Luxembourg : 55.5 n = 5 Other countries : 44.4 n = 4	64 560 (31 795 - 165 991)	411 (285–719)	Without evidence of exposure: 0 n = 0 Past : 44.4 n = 4 Ongoing : 55.6 n = 5	G1a : 66.7 n = 6
Others n = 22	40 (36–46)	Male: 68.2 n = 15 Female: 31.8 n = 7	Luxembourg : 36.4 n = 8 Other countries : 63.6 n = 14	535 (158 - 28 272)	637 (251–807)	Without evidence of exposure : 50 n = 11 Past : 9 n = 3 Ongoing : 41 n = 9	G1a: 66.7 n = 8 G1b: 16.7 n = 2 G3a : 16.7 n = 2

^a^ IQR, interquartile range

^b^ HCV, Hepatitis C Virus, 48 HCV genotypes available/56 HCV infection

^c^ HIV, Human Immunodeficiency Virus.

Preliminary interviews performed by the clinicians during the fisrt consultation of 32/68 (47%) PWID (24/32 belong to the B cluster and 8/32 did not belong to any clusters), newly diagnosed with HIV, revealed that 12 were homeless (37.5%), and that all were more likely to be infected in Luxembourg since they all described to inject only in Luxembourg. Importantly, 27/32 (84.4%) of these PWID, newly diagnosed with HIV, reported injecting frequently both cocaine and heroin (at least once a day).

In this regard, an increase in cocaine injection and in overall cocaine use for primary and secondary substance uses, reported by the national drug monitoring system RELIS, was observed between 2012 and 2015 from 53 to 63% (Table 2). Statistics collected routinely at SDCF corroborate this trend as the proportion of clients using cocaine and cocaine mixtures has been increasing from 5% in 2012 to 22% in 2016. Among the 77 HIV-infected PWID referred at the NSID, 59 PWID reported to be nationaly recorded in RELIS, 62% (36/59 drug users) to attend NSP/OST facilities and to be previously tested for HIV. These data suggest that most of the drug users newly infected with HIV were attending frequently the drug treatment facilities, were probably living in Luxembourg for years and counselled on the risks of infectious diseases transmission via not sterile injection paraphernalia by the drug treatment services.

**Table 2:** Primary and secondary cocaine use (valid %) in the nationally recorded drug users (RELIS [1]) and at the supervised drug consumption facility.

Year	RELIS recorded drug users						Supervised drug consumption rooms		
	Primary drug^a^		Secondary drug^b^		Total	n			
	*Cocaine IDU*^c^	*Cocaine non-IDU*^c^	*Cocaine IDU*^c^	*Cocaine* *non-IDU*^c^	Cocaine total		Cocaine	Cocaine mixtures	Cocaine total
**2012**	4% n = 13	9% n = 30	18% n = 60	22% n = 75	**53%** n = 178	336	3%	2%	**5%**
**2013**	6% n = 17	11% n = 32	13% n = 38	21% n = 60	**51%** n = 147	289	4%	3%	**7%**
**2014**	6% n = 16	14% n = 38	22% n = 60	19% n = 51	**61%** n = 165	271	6%	5%	**11%**
**2015**	8% n = 23	11% n = 32	20% n = 58	24% n = 70	**63%** n = 183	290	13%	9%	**22%**
**2016**							15%	7%	**22%**

^a^ The primary drug is defined according to EMCDDA guidelines as the drug that causes the client the most problems at the start of treatment. This is usually based on the request made by the clients and /or on the diagnosis.

^b^ Secondary drugs are those drugs used in addition to or as a substitute for the primary drug, and are substances that cause problems for the client and/or change the nature of the problem as assessed by the client and the caregiver.

^c^IDU, Injecting Drug Use.

Since the majority of new HIV diagnoses has described injecting drugs or sleeping at SDCF, we next performed an epidemiological study both at the SDCF and NSID including 170 PWID between October 2015 and December 2017. Among PWID, 17 reported injecting only cocaine, 90 reported injecting both cocaine and heroin, and 63 reporting injecting only heroin. Because the number of PWID injected only cocaine was small, subsequent analyses have compared PWID who injected cocaine +/- heroin with those who injected heroin only. Table 3 describes the sample characteristics stratified by current cocaine/heroin injection or heroin injection. The median age of all PWID at baseline was 41 years (IQR: 33.5–46), 107 (63%) participants reported cocaine injection use in the preceding six months (17 reported cocaine use alone and 90 reported both cocaine and heroin use); 63 participants were injecting heroin only. The univariate analysis showed that PWID injecting cocaine and/or heroin were younger (mean of 38 vs 44 years, p≤0.001), shared drugs more often (72 vs 51%, p≤0.01) (as an example, PWID often withdraw the drug from the same syringe in order to share equally the volume among several users) and were more frequently HIV positive at their last test (14.9 vs 4.8%, p≤0.05) than PWID injecting heroin only. In addition, they described more often to inject heroin or cocaine regularly (at least daily) than heroin users (88.7 vs 73%, p≤0.01), and reported more frequent piercing (38.3 vs 14.3%, p≤0.01). Although it was not statitically signficant (p = 0.07), a higher number of drug users injecting cocaine and heroin reported to share syringes as compared to PWID injecting heroin only (20.8 vs 9.4%).

**Table 3:** Characteristics of the 170 participants from the national supervised drug consumption facility stratified by cocaine injection (associated with or without heroin) versus heroin injection alone.

	Total (%)		Drug usage	
Characteristic		Cocaine +/- heroin	Heroin	p-value
Age				
Median (IQR) (n = 170)	41 [33.5–46]	38 [32–45]	44 [38–52]	0.0003
Age of first consumption				
Median (IQR) (n = 170)	17 [14–20]	17 [14–20]	17 [15–21]	0.44
Regular consumption (at least daily) (n = 169)				
Yes	140 (82.9%)	94 (88.7%)	46 (73.02%)	0.009
No	29 (17.16%)	12 (11.32%)	17 (26.98%)	
Under OST (n = 169)				
Yes	109 (64.5%)	67 (63.21%)	42 (66.67%)	0.64
No	60 (35.5%)	39 (36.79%)	21 (33.33%)	
Drug Sharing (usually) (n = 169)				
Yes	102 (64.15%)	73 (71.57%)	29 (50.88%)	0.009
No	57 (35.85%)	29 (28.43%)	28 (49.12%)	
Syringe sharing (usually) (n = 149)				
Yes	25 (16.78%)	20 (20.83%)	5 (9.43%)	0.07
No	124 (83.22%)	76 (79.17%)	48 (90.57%)	
Condom use (usually) (n = 140)				
Yes	68 (48.57%)	50 (54,35%)	18 (37.5%)	0.058
No	72 (51.43%)	42 (45,65%)	30 (62.5%)	
Prostitution (usually) (n = 170)				
Yes	21 (12.35%)	16 (14,95%)	5 (7.94%)	0.18
No	149 (87.65%)	91 (85,05%)	58 (92.06%)	
Prison (ever) (n = 170)				
Yes	74 (43,53%)	47 (43,93%)	27 (42.86%)	0.89
No	96 (56.47%)	60 (56,07%)	36 (57.14%)	
Piercing (n = 170)				
Yes	50 (29.41%)	41 (38,32%)	9 (14.29%)	0.0009
No	120 (70,59%)	66 (61,68%)	54 (85.71%)	
HIV + (n = 169)				
Yes	19 (11,24%)	16 (14,95%)	3 (4.84%)	0.045
No	150 (88,76%)	91 (85,05%)	59 (95.16%)	

IQR, interquartile range; OST, opioid substitution treatment

The unadjusted odd ratios derived from the univariate analysis (p≤0.05) are shown in Table 4. In the multivariable logistic regression analysis, adjusted odd ratios showed that age > piercing > drug sharing > regular consumption > remained independently associated with cocaine use (Table 4) but not HIV seropositivity (p = 0.086). Therefore, older individuals were less likely to consume both cocaine and heroin, whereas cocaine users were more likely to be younger, to have piercing, to share drugs, and to consume frequently.

**Table 4:** Unadjusted and adjusted odd ratios from the multivariable logistic regression analysis.

Characteristic		Odds ratio (OR)							
		Unadjusted OR (95%CI)				Adjusted OR (95%CI)			
		OR (N = 157)	95%CI		p-value	OR (N = 157)	95%CI		p-value
**Age**									
per year older		0.931	0.895	0.969	0.0004	0.944	0.905	0.985	0.0078
**Regular use**									
Yes vs No		3.273	1.394	7.682	0.0065	3.097	1.194	8.034	0.0201
**Drug sharing**									
Yes vs No		2.607	1.319	5.152	0.0058	2.488	1.167	5.306	0.0183
**Piercing**									
Yes vs No		4.403	1.813	10.694	0.0011	3.269	1.285	8.318	0.0129
**HIV +**									
Yes vs No		3.081	0.852	11.148	0.0863	1.650	0.411	6.629	0.4800

The reference is cocaine users, p < 0.05 for all unadjusted odd ratios

The analysis of Maximum Likelihood Estimates failed to link HIV seropositivity to cocaine and heroin users or to heroin users only. The sensitivity analysis included 3 more variables in the full multivariable model (Syringe sharing, Condom use, Prostitution). Manual backward variable selection lead to a final model with significant variables being age, piercing and drug sharing (S1 Table). Regular consumption was no more significant in this last model which may indicate a lower robust association with cocaine use.

## Discussion

Using different data sources, we confirm in the present work a recent HIV outbreak among PWID in Luxembourg mainly due to network transmission: (1) Phylogenetic analysis of sequences from all HIV-infected patients referred to NSID showed that the HIV transmission among PWID was concentrated in 2 two monophylogenetic clusters (46 of 77 PWID), (2) The RELIS database indicate the increase in cocaine supply both in drug treatment services and at SDCF, and (3) the epidemiologic analysis on drug consumption performed mainly at SDCF suggest that injecting cocaine might be a contributing factor related to HIV infection since this factor was significant in univariate analysis. Data routinely collected at SDCF showed the higher frequency of injections per day for cocaine users, leading to increased needs of sterile injection paraphernalia. Moreover, it is tempting to speculate that the introduction of cocaine injection contributed to a major change in psychosocial behavior challenging existing prevention measures against HIV infection. Our data suggest indeed that most of the newly diagnosed patients had knowledge on HIV transmission and prevention by attending NSP/OST facilities and being tested previously for HIV.

Since 2012, introduction of cocaine in Luxembourg had a major impact on the life-style and marginalisation of PWID. Change in psychosocial behavior combined with higher frequency of injections per day, described as well by cocaine addicts from SDCF, might have impacted on HIV transmission among cocaine injectors. We hypothesize that marginalized homeless drug users, having less access to NSP, and injecting often cocaine in combination with heroin [14] may have higher risk behaviors. This precarious behavior could have fostered HIV transmission among other drug users. The majority of the subtype B cluster was indeed identified at the SDCF in drug users living in the neighboring area of SDCF and sleeping in the emergency shelter. Stimulant use and high-risks behaviours were highly associated in recent outbreaks among PWID [5, 6, 9]. Cocaine is the predominant illicit stimulant used in Southern and Western European countries; however, injection of cocaine is less common. In Luxembourg, cocaine reached a high level as primary or secondary drug in the last 3 years. The inhalation mode is becoming a new trend of cocaine use while insufflation were only reported by 2% of SDCF users. Recent market indicators suggest a rise of cocaine availability in certain parts of Europe, and increasing seizures were reported since 2014 in France and Belgium [19]. No substitution treatement for cocaine misuse currently exists. Cocaine abuse facilitates violence and problems in decision-making [20] which may have a negative impact on established prevention and harm reduction measures.

More frequent daily injections in cocaine users compared to heroin users and its correlates in terms of risk behaviours might explain to some extend the observed increase in the HIV transmission rate [21]. From the multivariable analysis, piercing, regular consumption, drug sharing and age remained independently associated with cocaine use as compared to heroin use only. Syringe sharing was not frequently reported in both groups, potentially because the study included only PWID attending the SDCF. Interestingly, sharing of drugs was often described by cocaine users to inject more often and to avoid craving but this behaviour was generally not perceived as a risk transmission behaviour by recruited users. Moreover, the number of sterile syringes distributed via the national NSP programme decreased from 2004 to 2014 [15], at the onset of the HIV outbreak, although harm reduction offers have been further developped nationally, and a higher amount of syringes was potentially needed due to higher frequency of cocaine injections. Since 2015, the numbers of clean syringes distributed have been increasing moving Luxembourg towards a high NSP coverage country as compared to previous estimates showing medium coverage [22]. Ultimately, efforts should be pursued to further facilitate access to sterile injection paraphernalia and maintain a high national NSP coverage.

Two transmission clusters are driving the epidemic in PWID (46/77 PWID belong to the 2 clusters) with local HIV strains, in contrast to other European outbreaks [11]. In the phylogenetic analysis, highly divergent viruses appeared in the large HIV-1 subtype B cluster in a relative short time period High genetic diversity may be indicative of transmissions from different sources such as sharing of needles/serynges, drugs but also unsafe sex. Sexual transmission might have also contributed largely to the HIV outbreak in cocaine users [21, 23]. Cocaine users have a higher incidence of risky sexual behavior than non-users and cocaine use increases sexual appetence [23]. Interestingly, condom use was more frequently reported in cocaine users as compared to heroin users (50 individuals versus 18, respectively, p = 0.058). This might be explained by a higher sexual activity in younger cocaine addicts leading them to report more frequently the use of condom as compared to older heroin users. Females were highly represented in cocaine users (38%) and especially in newly infected PWID (33%), and reporting trading sex for drugs or money. Casualization and homelessness were also counfounding risk factors; both increased in parallell with cocaine injection use at the SDCF. Between 2014 and 2017, pragmatic data from SDCF showed that 37 different nationalities were represented: 30% of all clients had no social security coverage, and 28% declared to be homeless.

## Conclusions

A recent HIV outbreak was observed in PWID injecting cocaine, living in precarious situation in Luxembourg. Prevention offers including HIV testing have been reinforced in harm reduction facilities, outreach offers and SDCF. This might have contributed to some extend to the higher number of detected cases in 2015 and 2016. 56/68 PWID (82%) were under antiretroviral treatment and 50/68 (73%) had an undetectable viral load in december 2017; early access to antiretroviral therapy being actively used as a prevention measure to contain the outbreak. Outreach offers targeting injectors and marginalised drug users were further developped. Preexposure prophylaxis in active PWID might also be considered as an additional tool in the near future [24], especially for women and cocaine injectors for which no substitution therapy exists. Ultimately, the recurring of HIV infection in PWID in the recent years emphasises the added value of continuous drug monitoring systems in the detection of emerging drug use patterns. Early indicators of behaviour’s changes among drug users need to be recorded for the elaboration of fast and targeted responses.

## Supporting information

S1 Dataset HCV-UD dataset. Epidemiological information of 170 injector drug users interviewed at the SDCF between October 2015 and December 2017 using a standardized questionnaire including: demographic and social characteristics, drug use patterns, risk and harm reduction behaviours and infectious diseases status.
(XLSX)

S1 Table Adjusted odd ratios from the multivariable sensitivity analysis. (DOCX)
