# Supplementary material for: Injection of cocaine is associated with a recent HIV outbreak in people who inject drugs in Luxembourg
Source: PLoS One. 2019 May 16;14(5):e0215570. doi: 10.1371/journal.pone.0215570 (PMC6522034; doi:10.1371/journal.pone.0215570)
Supplement: S1 Table — (DOCX) [file pone.0215570.s002.docx]

**Supplemental Table 1. Adjusted odd ratios from the multivariable sensitivity analysis.**

|  | Full Model | | | |  | Final Model | | | |
| --- | --- | --- | --- | --- | --- | --- | --- | --- | --- |
| Characteristic | Adjusted OR (95%CI) | | |  |  | Adjusted OR (95%CI) | | |  |
|  | OR (N=120) | 95%CI | | p-value |  | OR (N=120) | 95%CI | | p-value |
| **age** |  |  |  |  |  |  |  |  |  |
| per year older | 0.931 | 0.882 | 0.983 | 0.0101 |  | 0.933 | 0.886 | 0.983 | 0.0266 |
| **Regular consumption** |  |  |  |  |  |  |  |  |  |
| Yes vs No | 1.560 | 0.503 | 4.836 | 0.4411 |  | 3.352 | 1.408 | 7.982 | 0.2213 |
| **Drug Sharing** |  |  |  |  |  |  |  |  |  |
| Yes vs No | 2.717 | 1.064 | 6.939 | 0.0367 |  | 3.659 | 1.219 | 10.984 | 0.2804 |
| **Piercing** |  |  |  |  |  |  |  |  |  |
| Yes vs No | 3.912 | 1.235 | 12.394 | 0.0204 |  |  |  |  |  |
| **HIV +** |  |  |  |  |  |  |  |  |  |
| Yes vs No | 0.964 | 0.201 | 4.616 | 0.9634 |  |  |  |  |  |
| **Condom use** |  |  |  |  |  |  |  |  |  |
| Yes vs No | 2.430 | 0.958 | 6.163 | 0.0616 |  |  |  |  |  |
| **Syringe sharing** |  |  |  |  |  |  |  |  |  |
| Yes vs No | 1.581 | 0.417 | 5.985 | 0.5003 |  |  |  |  |  |
| **Prostitution** |  |  |  |  |  |  |  |  |  |
| Yes vs No | 1.087 | 0.234 | 5.057 | 0.9156 |  |  |  |  |  |
